# Genetic Evidence for a Phosphorylation-Independent Signal Transduction Mechanism within the *Bacillus subtilis* Stressosome

**DOI:** 10.1371/journal.pone.0090741

**Published:** 2014-03-05

**Authors:** Tatiana A. Gaidenko, Chester W. Price

**Affiliations:** Department of Microbiology & Molecular Genetics, University of California Davis, Davis, California, United States of America; Loyola University Medical Center, United States of America

## Abstract

The stressosome is a 1.8 MDa cytoplasmic complex that controls diverse bacterial signaling pathways. Its role is best understood in *Bacillus subtilis*, where it activates the σ^B^ transcription factor in response to a variety of sharp environmental challenges, including acid, ethanol, heat or salt stress. However, details of the signaling mechanism within the stressosome remain uncertain. The core of the complex comprises one or more members of the RsbR co-antagonist family together with the RsbS antagonist protein, which binds the RsbT kinase in the absence of stress. As part of the response, RsbT first phosphorylates the RsbRA co-antagonist on T171 and then RsbS on S59; this latter event correlates with the stress-induced release of RsbT to activate downstream signaling. Here we examine the in vivo consequence of S59 phosphorylation in a model strain whose stressosome core is formed solely with the RsbRA co-antagonist and RsbS. A phosphorylation-deficient S59A substitution in RsbS blocked response to mild stress but had declining impact as stress increased: with strong ethanol challenge response with S59A was 60% as robust as with wild type RsbS. Genetic analysis narrowed this S59-independent activation to the stressosome and established that significant signaling still occurred in a strain bearing both the T171A and S59A substitutions. We infer that S59 phosphorylation increases signaling efficiency but is not essential, and that a second (or underlying) mechanism of signal transduction prevails in its absence. This interpretation nullifies models in which stressosome signaling is solely mediated by control of RsbT kinase activity toward S59.

## Introduction

Particular bacterial sensory modules often regulate activity of a variety of output domains to accomplish different signaling tasks. The 1.8 MDa stressosome complex appears widespread among the eubacteria, and its components are encoded in different genome contexts, implying it provides sensory function for diverse signaling pathways [1]–[3]. These components are best defined in *Bacillus subtilis*, where the stressosome controls activation of the general stress factor σ^B^ in response to rapidly increasing physical or chemical signals in the environment [4]–[6]. However, understanding of the mechanism by which the stressosome senses and conveys information remains limited. Here we use a genetic approach to assess the importance of a key phosphorylation event on a core stressosome protein, and as a result provide evidence for another, phosphorylation-independent route of information transfer within the complex.

Genetic and biochemical analysis indicates that the *B. subtilis* stressosome is formed from three different types of proteins: one or more members of the partially redundant RsbR co-antagonist family, the RsbS antagonist, and the RsbT serine-threonine kinase [7]–[11]. The four RsbR co-antagonists (RsbRA, RB, RC and RD) have different N-terminal, non-heme globin domains and more conserved C-terminal STAS (sulfate transporter/anti-sigma factor antagonist) domains, whereas the smaller RsbS antagonist comprises only a STAS domain [2], [12]. Twenty RsbR and ten RsbS dimers multimerize via these STAS domains to form the pseudo-icosahedral core of the stressosome, and in complexes formed in vitro the RsbT kinase appears to bind the surface of this structure at positions occupied by the RsbS antagonists [2]. The N-terminal domains of the RsbR dimers form outward projections from the stressosome surface and are thought to provide sensory input [2], but with the exception of YtvA – a distinct RsbR family member with an N-terminal, blue-light-sensing LOV domain [13]–[18] – there is no experimental evidence regarding how stress signals are sensed and conveyed to the core [19], [20].

**Figure 1:**
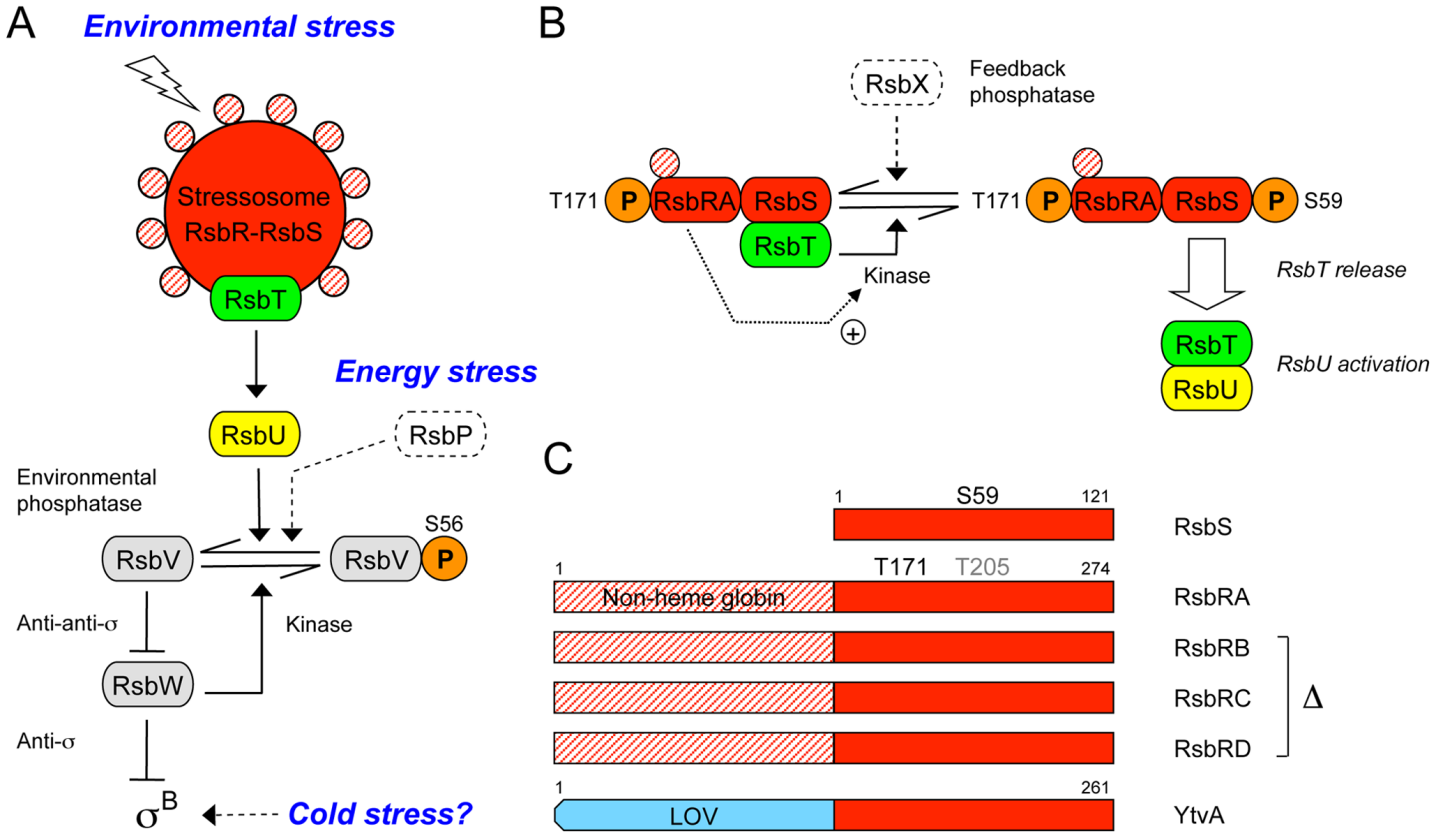
Stressosome and model of RsbRA-RsbS-RsbT activation. (A) Cytoplasmic stressosome complex (RsbR-RsbS-RsbT) controls activation of the environmental phosphatase (RsbU) in response to diverse physical and chemical signals. STAS domains of RsbR and RsbS form the stressosome core (red); N-terminal domains of RsbR are hypothetical sensors (red crosshatch); dissociable RsbT kinase (green) is positive activator of RsbU (yellow). RsbU removes the phosphoryl group (orange) from S56 on the RsbV anti-anti-σ, ultimately activating the σ^B^ stress factor. The energy phosphatase (RsbP) and the phosphatase-independent cold stress pathways are shown in dotted outline. Arrowheads indicate activation of protein targets or enzymatic reactions; T-headed lines indicate inhibition. (B) Model of stressosome control of RsbU activity. Stressosome core comprises partially redundant RsbRA, RB, RC, and RD co-antagonists (represented here as RsbRA) and the RsbS antagonist, which binds the RsbT kinase. In unstressed cells RsbT phosphorylates RsbRA on T171, facilitating subsequent activation of RsbT kinase activity toward RsbS (+ arrow). During the stress response RsbT phosphorylates RsbS on S59; RsbT is released to bind and activate RsbU. The RsbX feedback phosphatase (dotted outline) dephosphorylates RsbS-P. (C) RsbS comprises a single STAS domain (red rectangle), whereas the larger RsbRA has an N-terminal non-heme globin domain (red crosshatching) and C-terminal STAS domain (red). Phosphorylated S59 and T171 residues lie in the STAS domains; T205 (light grey) is phosphorylated only under extreme stress (see text). RsbRB, RC and RD are similar to RsbRA, with corresponding phosphorylated residues (not shown); these three paralogs were removed in some strains. YtvA is an RsbR family member that increases stressosome output in response to blue light, sensed by its N-terminal LOV (Light-Oxygen-Voltage) domain (blue). Figure modified from ref [20].

More is known about downstream signaling events. Stressosome output is represented by release of the RsbT kinase, which then binds and activates the RsbU environmental phosphatase by direct protein-protein interaction [7], [21], [22]. As part of this release, the RsbT kinase phosphorylates both RsbRA and RsbS on conserved residues within their STAS domains, both in vitro and in vivo [22]–[25]. The signaling model shown in Fig. 1 is supported by biochemical analysis, the phenotypes of null alleles as well as phosphorylation-deficient and phosphomimetic substitutions, and analysis of the phosphorylation state of key residues during the stress response [5]. Much of this in vitro and in vivo analysis, including structural analysis by hybrid methods [2], has made use of a minimal functional stressosome consisting of only the RsbRA, RsbS and RsbT proteins. Strains encoding such minimal stressosomes more readily reveal the effects of genetic alterations in *rsbRA* [8], [19], [20]. They also serve as useful models for the numerous bacterial species that lack multiple RsbR paralogs and possess only a single RsbRA-like protein [1], [3]. We therefore used strains encoding minimal stressosomes for the majority of experiments reported here.

According to the model shown in Fig. 1, T171 of RsbRA is phosphorylated even in unstressed cells; this event is thought to be an important prerequisite but does not by itself trigger the environmental stress response [8], [24]–[26]. Rather, T171 modification appears to facilitate subsequent phosphorylation of residue S59 on RsbS when stress is encountered. A negative charge at the S59 position decreases RsbS affinity for RsbT in pairwise binding experiments [3], [22] and results in RsbT release from the stressosome in vitro [7]. Correspondingly, either S59 phosphorylation or a phosphomimetic S59D substitution correlates with activation of the response in vivo [24], [27]. By contrast, T205 of RsbRA is thought to be phosphorylated only under extreme stress in order to inhibit signaling [25]. RsbX – a feedback phosphatase encoded in the neighborhood of the RsbRST cluster in *B. subtilis* and other bacteria – resets the system by removing the phosphate from S59 and T205 [1], [22], [25], [26].

Thus the RsbR co-antagonists and the RsbS antagonist have both negative and positive signaling roles, and these roles can be differentially affected by mutations within their structural genes. The known negative role of the RsbR co-antagonists is their required cooperation with RsbS to sequester RsbT in unstressed cells [7], [8]; null mutants missing all four RsbR paralogs or the single RsbS protein result in constitutive signaling in vivo [27], [28]. The presumed positive role of the RsbR co-antagonists is to stimulate RsbT kinase activity toward RsbS during the stress response [23], [26], which is thought to trigger the positive signaling function of RsbS; phosphorylation-deficient substitutions at RsbRA T171 or RsbS S59 cause diminished or nonexistent response in vivo [8], [27], depending on genetic context. In this regard, the S59A substitution in RsbS eliminates detectable phosphorylation in vitro [22], but its original phenotypic characterization suggested that S59A supports at least some signaling activity in an otherwise wild-type strain [27]. This implies that S59 phosphorylation is important but not essential for signaling. In accord with this result, more recent assays of the S59A substitution in model strains whose stressosomes were formed solely with the RsbRA or RsbRC co-antagonists found unexpectedly robust stress responses, indicating the existence of a signaling pathway independent from S59 phosphorylation [8], [19]. However, the nature of this S59-independent pathway has not been further explored, and it remains uncertain whether such signaling is indeed associated with the environmental branch of the regulatory network, or if it instead occurs via the distinct energy- or cold-signaling branches (Fig. 1).

Perhaps due to this uncertainty, prevailing models of stressosome signaling focus on how the phosphorylation state of S59 can be modulated by controlling RsbT kinase activity [2], [4], [24], [29], largely overlooking the observations that environmental stresses can activate σ^B^ in an S59A mutant. A further investigation of the influence of S59A on stress signaling therefore seemed warranted. We report here that the S59A substitution decreases signaling efficiency but does not prevent the stress response. Moreover, mutant analysis locates the S59-independent signaling mode to the environmental branch of the network, leading us to propose that it reflects the fundamental mechanism of signal transduction within the stressosome.

## Results

### Strains Deficient in S59 Phosphorylation Still Respond to Ethanol Stress

For our initial experiments investigating the effect of RsbS S59A on stress signaling, we used three strains whose stressosomes differed in their complement of RsbR family co-antagonists: one had the wild-type set of all four co-antagonists whereas the others had only one of the two principal co-antagonists, either RsbRA alone or RsbRB alone, together with the RsbS antagonist. Such strains activate σ^B^ equally well in response to ethanol stress, but their stressosomes exert different degrees of control over the unstressed output of the signaling network, with the wild type complex having the lowest basal output and thus the greatest control, RsbRB slightly less control, and RsbRA less still [8], [10]. As is the case for wild type, strains encoding only RsbRA or RsbRB nonetheless manifest an excess of stressosome core needed to bind the available RsbT, so these differences in basal level appear to reflect a fundamental property of the co-antagonist [10]. That is, when paired with RsbS each co-antagonist species could produce a complex with a characteristic affinity for RsbT or susceptibility to phosphorylation, or perhaps a different sensitivity to the activating signal, and thus would sequester RsbT with different efficiency in unstressed cells [10].

**Figure 2:**
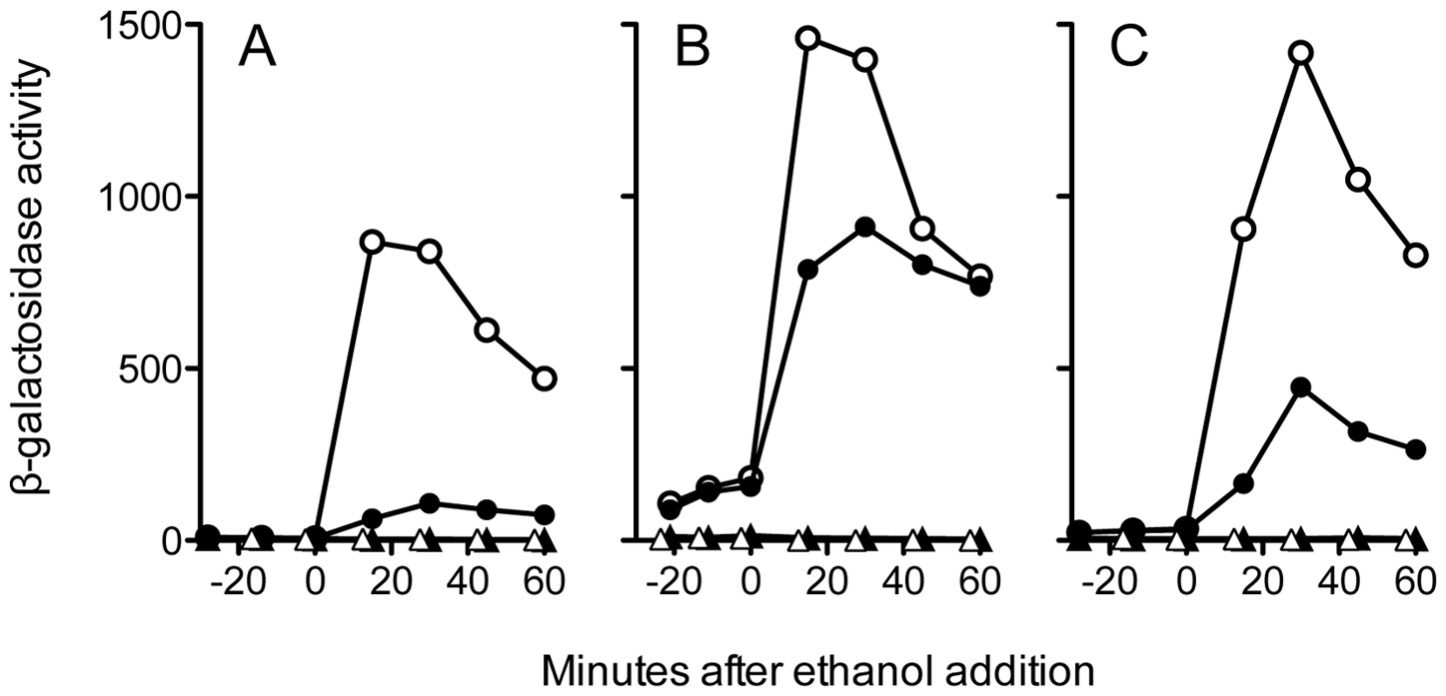
Strains bearing the S59A substitution retain significant stress activation. β-galactosidase accumulation from a σ^B^-dependent *ctc-lacZ* fusion, assayed in logarithmically growing cells either wild type for RsbS (open circles) or with the S59A substitution (closed circles), before and after 4% ethanol addition. In all three panels an RsbU null strain defective for the response served as the negative control (PB495, open triangles). (A) Wild type stressosome with all four co-antagonists and wild type RsbS (PB198, open circles); RsbS-S59A (PB470, closed circles); or RsbU null with RsbS-S59A (PB1274, closed triangles). (B) Minimal stressosome containing RsbRA as sole co-antagonist and wild type RsbS (PB1078, open circles); RsbS-S59A (PB1161, closed circles); or RsbU null with RsbS-S59A (PB1273, closed triangles). (C) Minimal stressosome containing RsbRB as sole co-antagonist and wild type RsbS (PB1255, open circles); RsbS-S59A (PB1256, closed circles); or RsbU null with RsbS-S59A (PB1275, closed triangles). Representative results are shown; in independent experiments S59A supported a response 13% as robust as wild type RsbS in the strain with all four co-antagonists (+/−2.0% SEM, n = 4); 63% in the strain with RsbRA alone (+/−2.1%, n = 7); and 33% in the strain with RsbRB alone (+/−1.3%, n = 3).

We first tested the effect of the S59A substitution in the strain with a full complement of RsbR family co-antagonists and found it decreased maximal response to ethanol stress about eight-fold (Fig. 2A); in four independent assays the mutant still retained 13% of the activity manifested by the strain with wild type RsbS. This 13% activation was eliminated in a strain bearing a null *rsbU* allele and thus lacking the RsbU environmental phosphatase: the *rsbS*-S59A *rsbU* double mutant had same low activity as the *rsbU* null control (Fig. 2A). This result indicates that the activation remaining in the S59A mutant originated in the environmental branch of the network and did not involve either the energy stress or cold stress pathways (Fig. 1), which are unaffected by loss of RsbU function [30], [31]. Because previous in vivo assays have shown that RsbU is incapable of transmitting signals of ethanol stress in the absence of input from RsbT [32], we draw the strong inference that the S59A-independent activation signal is conveyed via the recognized environmental signaling pathway upstream from RsbU, and not by RsbU itself or by a hypothetical independent pathway that converges on RsbU.

We then examined the effects of S59A in strains encoding simplified stressosomes. In the strain with stressosome complexes formed only with RsbRA, the RsbS mutant had 60% of parental activity (Fig. 2B), and with RsbRB about 30% (Fig. 2C). As was the case for the strain encoding all four RsbR co-antagonists, the heightened S59A-independent activation in the RsbRA or RsbRB strains was eliminated by the *rsbU* null (Fig. 2B and 2C). Moreover, the observation that the magnitude of the S59A-independent activation was significantly influenced by the co-antagonist complement of the stressosome further supports the conclusion that this activation is dependent upon the recognized signaling pathway upstream from RsbU.

### Significant Signaling can Occur in the Absence of both S59 and T171 Phosphorylation

The only known signaling element upstream from RsbU is the stressosome itself [5], whose core constituents are the RsbR paralogs and RsbS. To determine if a fully functional RsbRA co-antagonist was required for the S59A-independent activation mechanism, we assayed the effects of the T171A substitution in RsbRA. T171A eliminates the primary site of RsbRA phosphorylation [23], [24], [26] and manifests phenotypes of different severities, depending on the in vivo complement of stressosome components [8]. In a strain encoding all four RsbR co-antagonists and wild type RsbS, T171A effectively prevents signaling, decreasing ethanol stress response by 100 fold. By contrast, in a strain encoding only RsbRA and wild type RsbS, T171A decreases the response by 10–12 fold [8].

**Figure 3:**
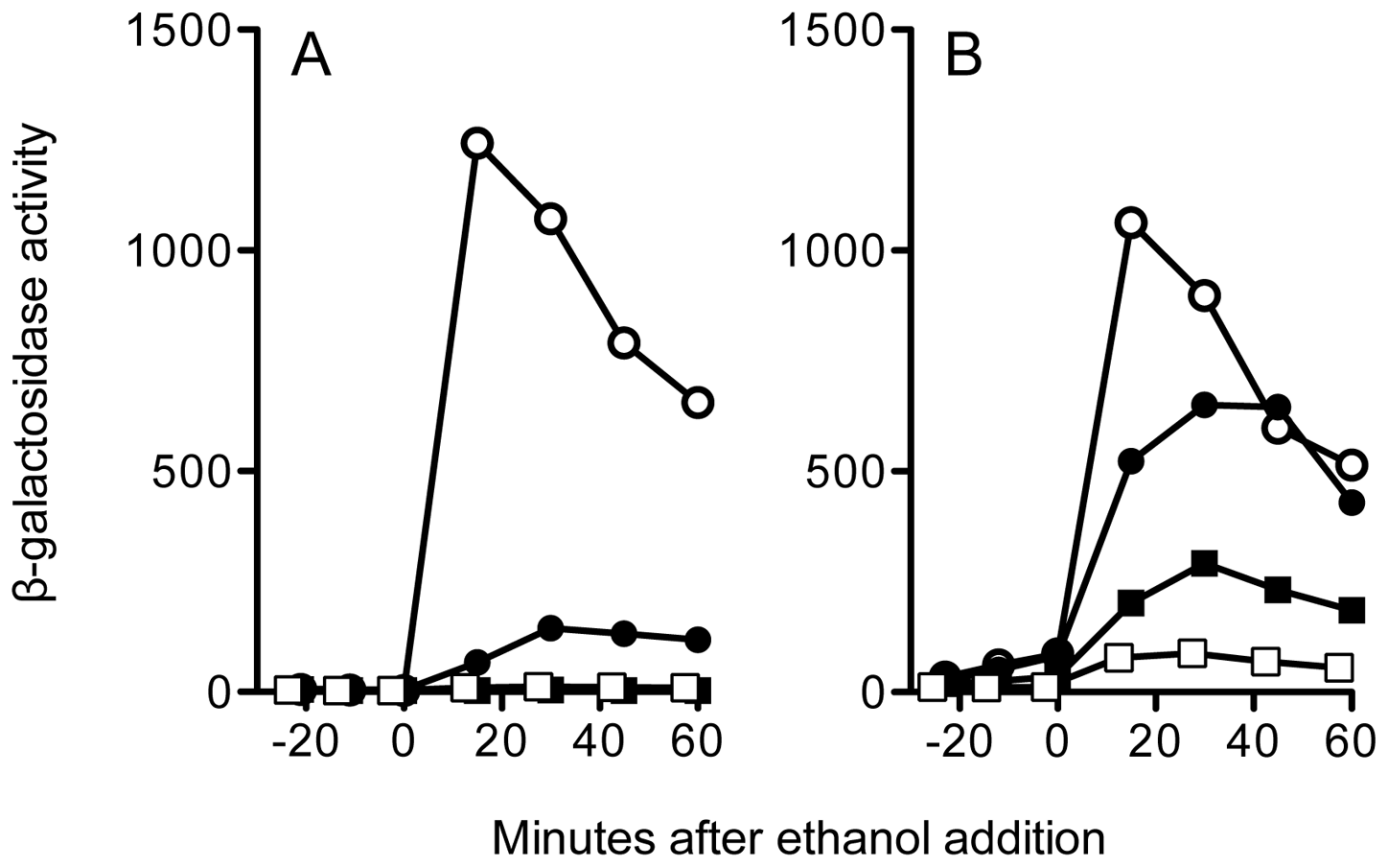
Positive epistasis between S59A and T171A in a model stressosome. β-galactosidase accumulation from a *ctc-lacZ* fusion in logarithmically growing cells, before and after 4% ethanol addition. (A) Wild type stressosome with all four co-antagonists and wild type RsbS and RsbRA (PB198, open circles); RsbS-S59A (PB470, closed circles); RsbRA-T171A (PB830, open squares); or RsbS-S59A together with RsbRA-T171A (PB1219, closed squares). (B) Minimal stressosome containing RsbRA as sole co-antagonist and wild type RsbS and RsbRA (PB1078, open circles); RsbS-S59A (PB1161, closed circles); RsbRA-T171A (PB1205, open squares); or RsbS-S59A with RsbRA-T171A (PB1190, closed squares). Representative results are shown; in independent experiments the T171A-S59A mutant manifested a response 0.2% that of wild type RsbRA and RsbS in the strain with all four co-antagonists (+/−0.03% SEM, n = 3); and 21% in the strain with RsbRA alone (+/−5.8%, n = 3).

In agreement with these previous results, we found that the T171A substitution eliminated signaling in an otherwise wild type strain encoding all four RsbR paralogs (Fig. 3A). T171A also blocked signaling in the RsbS S59A mutant, indicating that in the wild type stressosome the S59A-independent activity requires full RsbRA co-antagonist function. This result provides independent evidence that the activity originates in the environmental signaling pathway and does not involve either the energy or cold stress pathways. However, in this genetic background the strong T171A phenotype potentially obscured any epistatic interaction with S59A.

We therefore turned to the strain encoding the model stressosome formed from the RsbRA co-antagonist and RsbS. In this strain the T171A substitution only attenuated signaling about 12 fold, and produced an unexpected result when paired with S59A: the two substitutions manifested positive epistasis, with S59A in RsbS partially suppressing the phenotype of T171A in RsbRA (Fig. 3B). Positive (or alleviating) epistasis often results when two gene products act in concert within the same pathway [33]–[35]. In light of the extensive structural and biochemical data regarding interaction between RsbRA and RsbS [2], [7], [8], [10], [32], this additional genetic support is not surprising. However, if T171 and S59 phosphorylation were in fact key to the signaling mechanism [2], [4], [24], [29], the substantial stress activation evident in the T171A-S59A double mutant is unexpected (Fig. 3B). A structural basis for this effect is unlikely. When RsbRA and RsbS are incorporated into the pseudoatomic structure of the stressosome, both T171 and S59 lie on the exterior, solvent-exposed faces of their respective STAS domains, and alanine substitution at these residues would not impact surfaces thought to be important for STAS interaction within the core [2]. We therefore interpret our results to indicate that neither T171 nor S59 phosphorylation is required for signaling in the model stressosome.

### The S59-independent Route of Signal Transduction does not Involve the YtvA Light Sensor

We wished to further explore the nature of the S59-independent route of signaling within the stressosome. In this regard, Purcell et al. [36] proposed that some blue-light-sensing LOV domains may also have redox sensing capability within the bacterial cytoplasm. Notably, another protein associated with the *B. subtilis* stressosome is the blue-light-sensing YtvA regulator, an RsbR family member that carries an N-terminal LOV domain (Fig. 1). In contrast to the related RsbR co-antagonists, YtvA by itself does not appear capable of forming a stressosome with RsbS in vitro [37] and is only known to have a positive signaling role [11], [14], [15], [38]. Nonetheless, if its LOV domain were capable of redox as well as blue-light sensing, YtvA is a candidate for an S59-independent route of signaling, possibly detecting the secondary oxidative stress elicited by ethanol challenge [39].

**Figure 4:**
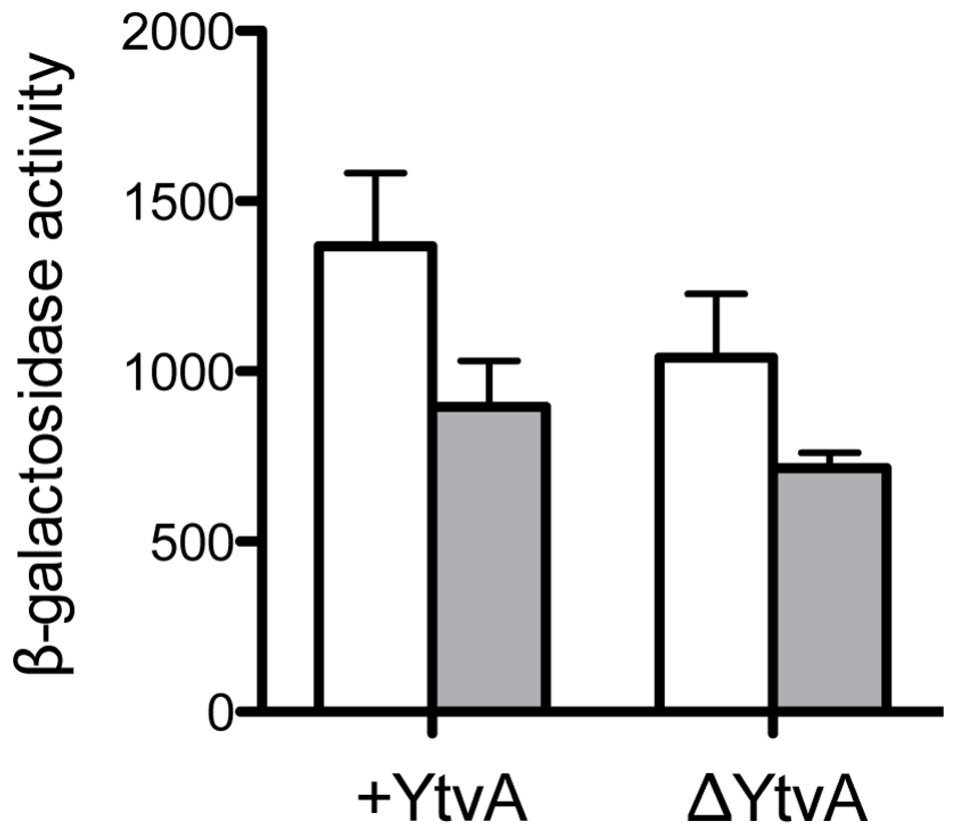
S59-independent activation does not require the YtvA blue-light sensor. Peak β-galactosidase accumulation from a *ctc-lacZ* fusion after 4% ethanol stress, in the presence or absence of YtvA (+YtvA or ΔYtvA). Strains encoded a minimal stressosome containing RsbRA as sole co-antagonist together with either wild type RsbS (PB1078 for +YtvA or PB1085 for ΔYtvA; open bars) or RsbS bearing the S59A substitution (PB1161 or PB1272; shaded bars). Error bars represent range in two independent assays.

To test the involvement of YtvA in ethanol stress signaling, we introduced a null *ytvA* allele into the parental and S59A mutant strains with RsbRA as the only co-antagonist. As shown in Fig. 4, loss of YtvA function reduced ethanol response of both the parent and the S59A mutant by a similar amount (20–25%), as expected for loss of a common positive regulator. However, the S59A mutant manifested the same significant fraction of the parental ethanol response in either the presence or absence of YtvA. We conclude that the S59A-independent route of signal transmission does not depend on YtvA.

### RsbS Phosphorylation Increases Response Efficiency

**Figure 5:**
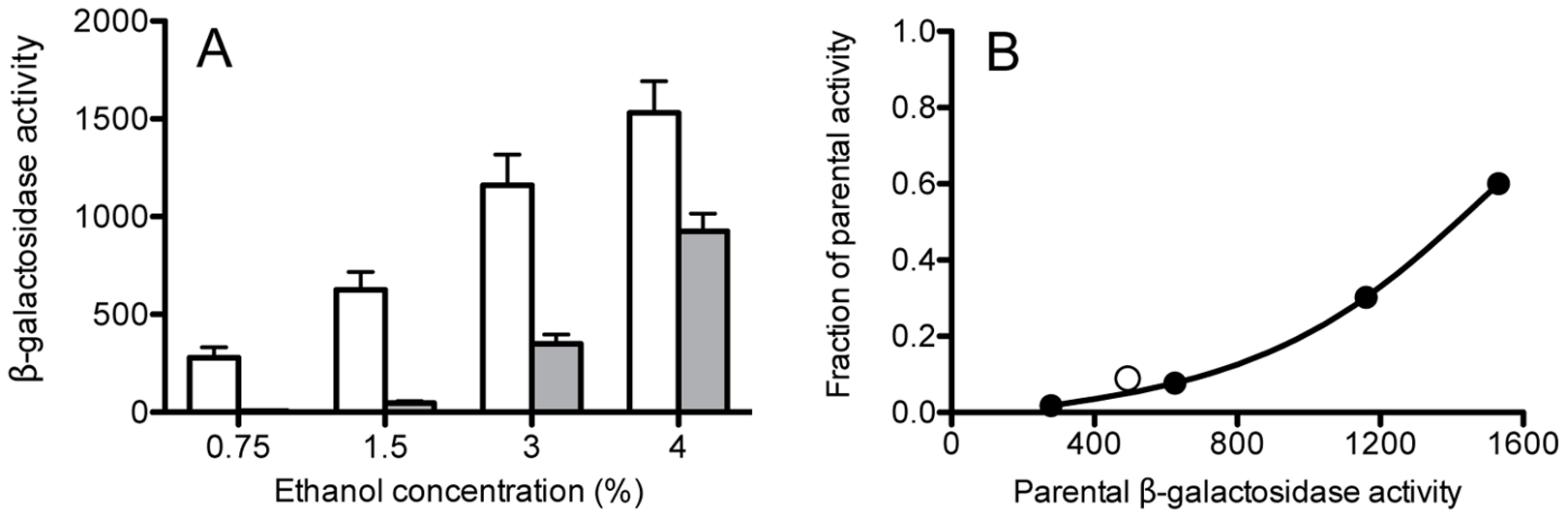
Ability to phosphorylate S59 increases stressosome sensitivity. (A) Peak β-galactosidase accumulation from a *ctc-lacZ* fusion after addition of different concentrations of ethanol. Strains encoded a minimal stressosome containing RsbRA as sole co-antagonist and either wild type RsbS (PB1078; open bars) or RsbS bearing the S59A substitution (PB1161; shaded bars). Error bars represent range in two independent assays. (B) Closed symbols indicate fraction of parental activity manifested by S59A mutant at each ethanol concentration (data from A); open symbol indicates fraction at 0.3 M NaCl (see text).

The results shown in Figures 2–4 were obtained in response to 4% ethanol stress. We next examined the effect of S59A on response to ethanol challenge over a range of lesser concentrations, using the strain encoding only the prototype co-antagonist, RsbRA [32]. As shown in Fig. 5A, S59A almost completely blocked response to a mild 0.75% ethanol stress, but its influence decreased significantly as ethanol challenge increased. This effect is readily apparent in Fig. 5B, which plots the data from Fig. 5A as the fraction of parental activity retained by the S59A mutant relative to the peak stress response of the parental strain. We infer from these results that S59 phosphorylation is critical for the transmission of low amplitude signals but becomes less significant as signal strength grows.

We attempted a parallel experiment with increasing salt stress. However, concentrations above 0.3 M NaCl had secondary effects that interfered with cell growth (data not shown). We were therefore unable to achieve responses of the magnitude necessary for full comparison to the ethanol data. However, the 0.3 M NaCl experiment did yield a potentially interesting result: the maximum response of the parental strain was 472 units (+/−SEM of 38; n = 3) whereas that of the S59A mutant was 28 units (+/−SEM of 9; n = 3), for a fractional response of 0.06. When this additional point was plotted as the open symbol in Fig. 5B, it fell near the line representing the ethanol data. This suggests that the effect of S59 phosphorylation is independent from the primary stress that elicits the response.

## Discussion

Most existing models of stressosome operation have focused on phosphorylation of RsbS S59 as the key signaling event within the stressosome complex [2], [4], [24], [29]. A presumed conformational change in the RsbR-RsbS core subunits has been suggested to influence activity of the RsbT kinase toward RsbS, either by allosteric control of kinase activity or by unmasking substrate residues within the core [2]. Along these lines, a recent computational model of stressosome operation was said to support the allosteric control of RsbT activity by phosphorylated RsbRA [29]. An acknowledged shortcoming of this model was its inability to fully replicate the phosphorylation kinetics of the in vivo study on which it was partly based. The in vivo study found that a rapid, post-stress increase in S59-phosphorylated RsbS occurred prior to a modest increase in T171-phosphorylated RsbRA [24], whereas in the computational model the increase in phosphorylated RsbS necessarily lagged the increase in phosphorylated RsbRA [29]. An unacknowledged shortcoming of the computational model was that another in vivo study, using a different growth medium, found no post-stress increase in T171-phosphorylated RsbRA at all [25]. These anomalies suggest that stress-dependent modulation of T171 phosphorylation does not well explain signal transmission within the stressosome, and that other mechanisms must play an important role.

In strong support of this view, the genetic results presented here speak against the prevailing phosphorylation models in their simplest forms. Stressosomes composed solely of mutant RsbRA and RsbS proteins, with phosphorylation-deficient T171A and S59A substitutions, were nonetheless capable of transmitting environmental stress signals in vivo (Fig. 3B). In vitro no other phosphorylation sites were detectable in the T171A-T205A mutant form of RsbRA or the S59A mutant form of RsbS [22], [23], and in vivo there is likewise no evidence for any additional sites on either protein [24], [25], [40]. Because in vivo phosphorylation of T205 primarily occurs under stress conditions more severe than we used here [24], [25], and this modification appears to suppress rather than activate signaling, for the purposes of our study its effects can be ignored. We therefore infer that significant in vivo activation of the stressosome can occur in the absence of both RsbRA and RsbS phosphorylation.

Our demonstration of a phosphorylation-independent signaling mechanism chiefly relied on a strain encoding a widely-used model stressosome in which RsbRA was the sole co-antagonist. This strain serves as an archetype for those bacterial species whose genomes encode only a single RsbR co-antagonist [1], the best characterized of which most closely resembles RsbRA [3]. However, we also extended these findings to show that RsbS phosphorylation was partly dispensable in two other strains (Fig. 2), one of which had wild-type stressosomes comprising all four co-antagonists [8], [9]. Our results should therefore be broadly applicable to stressosome operation in other bacterial signaling pathways, whether these stressosomes have but a single RsbR co-antagonist, as is the norm, or multiple co-antagonists, as is the case for *B. subtilis* and several other bacteria [1].

The existence of an RsbS-independent signaling role for RsbRA was initially suggested by the genetic epistasis test of Kim et al. [8], who found that the loss-of-signaling phenotype caused by the T171A substitution in RsbRA could not be fully reversed by the phosphomimetic S59D substitution in RsbS. This result points to a signaling role for RsbRA independent from its recognized ability to influence the rate of RsbS phosphorylation by RsbT [23], [26]. Here we confirm an RsbS-independent role by another means: a phosphorylation-deficient S59A substitution in RsbS decreased but did not completely block signaling, having the effect of altering the dose-response curve to require greater input for a comparable output (Fig. 5). This raises the intriguing possibility that S59 phosphorylation is an evolutionary addition that overlays a primordial signaling mechanism, increasing its sensitivity by increasing the dissociation rate of RsbT.

Based on the results reported here, we conclude that the stressosome manifests two mechanisms of signal transduction in response to acute environmental stress. We further suggest that these two mechanisms share an underlying commonality: a hypothetical signal-induced shift in conformational equilibria within the stressosome core [2]. One mechanism is dependent on RsbS phosphorylation and likely reflects RsbRA enhancement of RsbT kinase activity toward RsbS. This enhancement may involve active stimulation of the kinase or an unmasking of the RsbS substrate within the core; either could be elicited by the hypothetical conformational shift [2]. The other mechanism is independent of RsbS phosphorylation and its molecular basis is presently unknown. However, we propose that the same conformational shift can trigger RsbT release even in the absence of RsbS phosphorylation, albeit less efficiently. In this view, the signal-induced conformational shift is the fundamental signaling response, which then secondarily elicits activation of the RsbT kinase. Our genetic experiments with minimal stressosomes have uncovered this fundamental response, and offer a potential avenue for its exploration.

## Materials and Methods

### Bacterial Strains and Genetic Methods

*B. subtilis* strains shown in Table 1 are derivatives of PB2, a 168 Marburg strain originally obtained from Patrick Piggot [41]. Plasmids are shown in Table 2. Strain constructions employed two-step allele replacement [44], standard recombinant methods [45], and natural transformation [46]; all were confirmed by sequencing the coding regions of interest. A single-copy, *ctc-lacZ* transcriptional fusion provided an indirect measure of σ^B^ activity [42].

**Table 1:** *Bacillus subtilis* strains.

Name	Genotype or description	Construction^a^
PB2	*trpC2*	Marburg strain [41]
PB198	*amyE*::*ctc-lacZ trpC2*	[42]
PB470	*rsbS* S59A *amyE*::*ctc-lacZ trpC2*	[27]
PB491	*rsbRAΔ1 amyE*::*ctc-lacZ trpC2*	[32]
PB495	*rsbUΔ2 amyE*::*ctc-lacZ trpC2*	[31]
PB830	*rsbRA* T171A *amyE*::*ctc-lacZ trpC2*	[8]
PB1078	*rsbRBΔ2 rsbRCΔ1*::*ery rsbRDΔ2 amyE*::*ctc-lacZ trpC2*	[19]
PB1085	*rsbRBΔ2 rsbRCΔ1*::*spc rsbRDΔ2 ytvAΔ1::ery amyE*::*ctc-lacZ trpC2*	[19]
PB1161	*rsbS* S59A *rsbRBΔ2 rsbRCΔ1*::*ery rsbRDΔ2 amyE*::*ctc-lacZ trpC2*	[19]
PB1190	*rsbS* S59A *rsbRA* T171A *rsbRBΔ2 rsbRCΔ1*::*ery rsbRDΔ2 amyE*::*ctc-lacZ trpC2*	pTG6027→PB1161^b^
PB1205	*rsbRA* T171A *rsbRBΔ2 rsbRCΔ1*::*ery rsbRDΔ2 amyE*::*ctc-lacZ trpC2*	pTG6027→PB1078^b^
PB1219	*rsbRA* T171A *rsbS* S59A *amyE*::*ctc-lacZ trpC2*	pTG6027→PB470^b^
PB1254	*rsbRAΔ1 rsbRDΔ2 amyE*::*ctc-lacZ trpC2*	pTG5943→PB491^b^
PB1255	*rsbRAΔ1 rsbRCΔ1*::*ery rsbRDΔ2 amyE*::*ctc-lacZ trpC2*	pSA82^c^→PB1254
PB1256	*rsbS* S59A *rsbRAΔ1 rsbRCΔ1*::*ery rsbRDΔ2 amyE*::*ctc-lacZ trpC2*	pTG6009→PB1255^b^
PB1271	*rsbS* S59A *rsbRBΔ2 rsbRCΔ1*::*spc rsbRDΔ2 amyE*::*ctc-lacZ trpC2*	pEr::Sp^c^→PB1161
PB1272	*rsbS* S59A *rsbRBΔ2 rsbRCΔ1*::*spc rsbRDΔ2 ytvAΔ1*::*ery amyE*::*ctc-lacZ trpC2*	pSA68^c^→PB1271
PB1273	*rsbS* S59A *rsbRBΔ2 rsbRCΔ1*::*ery rsbRDΔ2 rsbUΔ3 amyE*::*ctc-lacZ trpC2*	pTG6110→PB1161^b^
PB1274	*rsbS* S59A *rsbUΔ3 amyE*::*ctc-lacZ trpC2*	pTG6110→PB470^b^
PB1275	*rsbS* S59A *rsbRAΔ1 rsbRCΔ1*::*ery rsbRDΔ2 rsbUΔ3 amyE*::*ctc-lacZ trpC2*	pTG6110→PB1256^b^

a Arrow indicates transformation from donor to recipient.

b Two-step allele replacement.

c Linearized plasmid.

**Table 2:** Plasmids for strain construction.

Plasmid	Relevant feature	Reference
pEr::Sp	Converts *ery* to *spc*	[43]
pSA68	*ytvAΔ1*::*ery* in pUC19 integrative plasmid	[28]
pSA82	*rsbRCΔ1*::*ery* in pUC19 integrative plasmid	[28]
pTG5916	*Nde*I site converted to I-*Sce*I in pUS19 integrative plasmid	[19]
pSS4332	Expresses I-*Sce*I for two-step allele replacement (pTG5916 vectors)	[44]
pTG5943	*rsbRDΔ2* in pTG5916	[19]
pTG6009	*rsbS* S59A in pTG5916	[19]
pTG6027	*rsbRA* T171A in pTG5916 (ACC→GCC)	This work
pTG6110	*rsbUΔ3* in pTG5916 (codons 12–331 deleted)	This work

### β-galactosidase Accumulation Assays

Assays were conducted as described previously [19]. Cultures were grown at 37 C in shake flasks containing buffered Luria broth lacking salt, and with moderate white light illumination (3 to 4 µmol m^−2 ^s^−1^). This illumination saturated the blue light-sensing positive regulator YtvA to establish a constant effect on assay results [47]. Unstressed samples were taken during early exponential growth up to a cell density of 20 absorbance units (Klett-Summerson colorimeter equipped with a number 66 transmission filter); ethanol stress was then imposed at the final concentrations indicated in the figures. Samples were treated as described by Miller [48], but with activity defined as Δ*A*_420_×1,000 min^−1 ^mg protein^−1^ (Protein Assay Reagent; Bio-Rad Laboratories, Hercules, CA). Stress activation was calculated as maximal post-stress activity minus basal activity just prior to stress addition.
